# Dual antithrombotic therapy and gastroprotection in atrial fibrillation: an observational primary care study

**DOI:** 10.3399/BJGPO.2022.0048

**Published:** 2022-10-19

**Authors:** Charis Xuan Xie, John Robson, Crystal Williams, Chris Carvalho, Stuart Rison, Zahra Raisi-Estabragh

**Affiliations:** 1 Wolfson Institute of Population Health, Barts and The London School of Medicine and Dentistry, Queen Mary University of London, London, UK; 2 North East London Integrated Care System, Unex Tower, London, UK; 3 Barts Heart Centre, St Bartholomew’s Hospital, Barts Health NHS Trust, London, UK; 4 William Harvey Research Institute, National Institute for Health and Care Research Barts Biomedical Research Centre, Queen Mary University London, London, UK

**Keywords:** dual antithrombin therapy, anticoagulation, antiplatelets, atrial fibrillation, cardiovascular diseases, gastroprotection, bleeding risk, population health

## Abstract

**Background:**

Patients with both atrial fibrillation (AF) and cardiovascular disease (CVD) may receive dual antithrombotic therapy (DAT) with both an anticoagulant and ≥1 antiplatelet agents. Avoiding prolonged duration of DAT and use of gastroprotective therapies reduces bleeding risk.

**Aim:**

To describe the extent and duration of DAT and use of gastroprotection in a primary care cohort of patients with AF.

**Design & setting:**

Observational study in 1.2 million people registered with GPs across four east London clinical commissioning groups (CCGs), covering prescribing from January 2020–June 2021.

**Method:**

In patients with AF, factors associated with DAT prescription, prolonged DAT prescription (>12 months), and gastroprotective prescription were characterised using logistic regression.

**Results:**

There were 8881 patients with AF, of whom 4.7% (*n* = 416) were on DAT. Of these, 65.9% (*n* = 274) were prescribed DAT for >12 months and 84.4% (*n* = 351) were prescribed concomitant gastroprotection. Independent of all other factors, females with AF were less likely to receive DAT than males (odds ratio [OR] 0.61, 95% confidence interval [CI] = 0.49 to 0.77). Similarly, older (aged ≥75 years) individuals (OR 0.79, 95% CI = 0.63 to 0.98) were less likely to receive DAT than younger patients. Among those with AF on DAT, pre-existing CVD (OR 3.33, 95% CI = 1.71 to 6.47) and South Asian ethnicity (OR 2.70, 95% CI = 1.15 to 6.32) were associated with increased gastroprotection prescriptions. Gastroprotection prescription (OR 1.80, 95% CI = 1.01 to 3.22) was associated with prolonged DAT prescription.

**Conclusion:**

Almost two-thirds of patients with AF on DAT were prescribed prolonged durations of therapy. Prescription of gastroprotection therapies was suboptimal in one in six patients. Treatment decisions varied by sex, age, ethnic group, and comorbidity. Duration of DAT and gastroprotection in patients with AF requires improvement.

## How this fits in

Patients with AF and CVD may be treated with DAT. Associated bleeding risk may be reduced using gastroprotective therapies and by avoiding prolonged DAT durations. In this large population-based cohort of patients with AF, among those on DAT, two-thirds of patients received treatment for excessive durations and use of gastroprotection therapies was suboptimal. Treatment decisions varied by sex, age, ethnic group, and comorbidity. The findings highlight urgent need for directed strategies to optimise medication management of patients with AF treated with DAT.

## Introduction

Ageing populations^1^ are leading to greater burde from chronic age-related illnesses, among which AF and coronary artery disease (CAD) are the most common CVDs.^2,3^ These conditions share multiple risk factors and commonly co-exist, presenting clinical challenges.^4^

Patients with CAD may be prescribed antiplatelets for event prevention in stable disease.^5^ In the setting of an acute coronary syndrome or after percutaneous coronary intervention (PCI), there is strong evidence for dual antiplatelet therapy to prevent myocardial infarction and, for those undergoing PCI, stent thrombosis.^6^ High-risk patients with chronic stable CAD also warrant antiplatelet therapy to prevent myocardial infarction or after elective PCI to prevent stent thrombosis.^6^ The typical duration of dual antiplatelet therapy after PCI is 12 months; however, there is increasing evidence to suggest that shorter durations of therapy are adequate for newer-generation drug-eluting stents and may be considered for patients at high bleeding risk.^6^

Treatment decisions are further complicated in patients with CAD and co-existent AF, who have an indication for anticoagulation to prevent thromboembolic stroke.^4^ In these individuals, a short period (1–3 months) of 'triple therapy' (combining dual antiplatelet therapy with anticoagulation) may be recommended, followed by DAT, comprising a single antiplatelet agent and an anticoagulant, for a further 3–11 months.^6^ Similar clinical challenges are encountered in patients with ischaemic cerebrovascular events with requirement for antiplatelet therapy; these individuals often have co-existent AF, which not infrequently has precipitated the cerebral event. Although durations of DAT vary, it is very rarely indicated for >1 year and for many the appropriate duration is <6 months.^4^

Concomitant use of multiple antithrombotic therapies is accompanied by increased risk of serious bleeding complications, most commonly upper gastrointestinal bleeding (UGIB). Routine use of gastroprotection therapies and avoiding excessive durations of DAT are important considerations for minimising DAT-related bleeding complications.^5–7^ However, the frequency with which such simple strategies are adopted in clinical practice is unclear. As most patients with stable chronic diseases are discharged to the community, it is important for both primary care and hospital clinicians to be vigilant to these issues.

UGIB is the commonest cause of hospital admission for an adverse drug-related event of which antiplatelet use, anticoagulant use, and particularly the combination of the two in conjunction with older age are substantive causes.^8^ A variety of evidence and guidelines now recommend proton pump inhibitors (PPIs) in people at high risk of bleeding on antiplatelet therapy, particularly in combination with anticoagulant therapy.^6,9–12^

The present study sought to evaluate factors associated with suboptimal bleeding risk mitigation in patients with AF using routine health data in a community setting. This work is particularly timely as the new national Network Contract Directed Enhanced Service Investment and Impact Fund 2020–2021 addresses medicines safety area and includes dual anticoagulant and antiplatelet therapy as a quality indicator for GPs in England.^13^

A population-based study of patients with AF is presented, describing demographic, physiologic, and clinical factors associated with DAT, and, among those on DAT, prolonged DAT (>12 months) and the prescription of gastroprotective therapies.

## Method

### Setting and study population

The study population included individuals registered with a GP in one of four east London CCGs (Waltham Forest, City and Hackney, Tower Hamlets, and Newham), covering a total of 1.2 million people. This population comprises individuals who are, on average, younger, more ethnically diverse, and less affluent than the general UK population.^14^ The CCGs have above national average performance in cardiovascular risk management.^14^ The study was limited to adults aged ≥18 years. Individuals were excluded who were registered with GPs for <12 months from the date of data extraction. Individuals were included with a record of diagnosed AF or atrial flutter, as per standardised SNOMED-CT codes (see Supplementary Table S1). Patients with a record of 'Atrial fibrillation resolved' were excluded.

### Data source

Deidentified health data were provided by the Clinical Effectiveness Group at Queen Mary University of London, who centrally extracted pseudonymised data from the general practice systems (EMIS) in June 2021, covering prescribing in the period from January 2020–June 2021. Data analysis was conducted in July 2021. Cases, demographic, and other risk factors and medication were identified using SNOMED-CT codes and routinely collected data.

### DAT and gastric protection medications

DAT was defined as concomitant prescription of ≥1 antiplatelet medications (aspirin, clopidogrel, dipyridamole, prasugrel, and ticagrelor) and an anticoagulant (warfarin, phenindione, acenocoumarol, apixaban, dabigatran, edoxaban, and rivaroxaban) within the preceding 6 months. Individuals prescribed DAT for >12 months were identified using an algorithm identifying prescription of both an anticoagulant and an antiplatelet medicine at both 18 months and within 6 months before data extraction. Prescription of gastroprotection medications in the preceding 6 months was additionally noted, specifically, PPIs (pantoprazole, rabeprazole, omeprazole, and esomeprazole), or H2-receptor antagonists (H2RAs: ranitidine, cimetidine, and famotidine).

### Risk factor indicators

Two age categories were considered of above and below 75 years, sex was defined as male or female, and five ethnic groups were considered aligned with the Office for National Statistics (ONS) 2001 census ethnic group classification:^15^ 1) White (British, Irish, any other White background); 2) Black (Caribbean, African, any other Black background); 3) South Asian (Indian, Pakistani, Bangladeshi, any other Asian background); 4) other ethnic groups including mixed ethnic groups and Chinese; and 5) those with no record of coded ethnic group.^16^ Deprivation was measured by the Index of Multiple Deprivation (IMD) 2015 and individuals were grouped into locally derived quintiles, from least (1) to most (5) deprived.^17^

Smoking history was classified as current smoker, ex-smoker, or never smoked (ever recorded). Body mass index (BMI) was directly extracted or calculated from records of height and weight. BMI was categorised into World Health Organization categories of obesity: <20 kg/m^2^ (underweight); 20–29 kg/m^2^ (reference level); and ≥30 kg/m^2^ (obese). Systolic blood pressure was extracted from recorded office measurements and classified into above and below 140 mmHg. All characteristics considered were based on the latest record within the past 3 years.

The following conditions were included, selected on their likely influence on clinical decisions regarding antithrombotic and gastroprotection therapies: ischaemic heart disease (IHD), stroke or transient ischaemic attack (TIA), diabetes (type 1 or type 2), chronic kidney disease (CKD), and previous UGIB. Disease definitions were based on relevant SNOMED-CT codes (see Supplementary Table S1).

### Statistical analysis

Statistical analyses were conducted using Stata (version 17.0). The association of clinical, physiological, and demographic factors with DAT prescription were estimated among patients with AF. Logistic regression models were used with DAT status (yes or no) set as the model outcome and participant characteristics set as the exposures of interest. Additionally, the study examined, among patients with AF on DAT, factors associated with prescription of gastroprotective therapy using logistic regression models, setting the model outcome as gastroprotection (yes or no) and inserting participant characteristics as the exposures of interest. Analyses were performed first in univariable and then in multivariable models with mutual adjustment for all exposures (age, sex, ethnic group, deprivation, CVD [IHD, stroke, or TIA], diabetes or stage 3–5 CKD, UGIB, systolic blood pressure, smoking, and BMI). Subgroup analysis was performed in patients prescribed DAT for 6 months and >12 months. Missing fields were treated as a separate category, ‘unknown’, in the univariable and multivariable logistic regression models without losing this information. Where the number of patients with missing data was very small (that is, <5) these patients were excluded from the model and indicated in the footnotes. The study reports ORs with 95% CIs and the corresponding *P*-values. Statistical significance level was set at 5%. The study conformed to the STROBE criteria.^18^

## Results

### Sample characteristics

A total of 8881 patients with AF were included in the analysis (Table 1 and Supplementary Figure S1). Almost half the patients (*n* = 4300/8881, 48.4%) were aged ≥75 years. There were slightly more males than females (*n* = 5024/8881, 56.6%). The degree of ethnic diversity was greater than in the national UK population with 36.6% (*n* = 3253/8881) of patients from ethnic minority backgrounds, most commonly from Black (*n* = 1186/8881, 13.4%) and South Asian (*n* = 1069/8881, 12.0%) ethnic groups. There was high prevalence of comorbidities, with diabetes or CKD recorded in 49.4% (*n* = 4389/8881) and CVD (IHD, stroke, or TIA) in 35.8% (*n* = 3177/8881). There was a record of obesity (BMI ≥30 kg/m^2^) in 33.8% (*n* = 3002/8881) of individuals. The sample included 8.9% (*n* = 792/8881) current smokers and 30.9% (*n* = 2742/8881) ex-smokers. Latest recorded systolic blood pressure measurement was ≥140 mmHg for 14.2% (*n* = 1262/8881) of patients.

**Table 1.** Baseline patient characteristics of individuals with AF stratified by DAT prescription

Characteristic	Whole sample (*N* = 8881), *n* %	Not on DAT (*n* = 8465), *n* %	On DAT (*n* = 416), *n* %
**Sex**			
Male	5024 (56.6)	4738 (56.0)	286 (68.8)
Female	3857 (43.4)	3727 (44.0)	130 (31.3)
**Age group, years**			
18–74	4581 (51.6)	4364 (51.6)	217 (52.2)
≥75	4300 (48.4)	4101 (48.4)	199 (47.8)
**Ethnicity**			
White	5628 (63.4)	5406 (63.9)	222 (53.4)
South Asian	1069 (12.0)	976 (11.5)	93 (22.4)
Black	1186 (13.4)	1137 (13.4)	49 (11.8)
Unknown^a^	998 (11.2)	946 (11.2)	52 (12.5)
**IMD quintile**			
1 (least deprived)	1782 (20.1)	1716 (20.3)	66 (15.9)
2	1784 (20.1)	1705 (20.1)	79 (19.0)
3	1765 (19.9)	1675 (19.8)	90 (21.6)
4	1774 (20.0)	1682 (19.9)	92 (22.1)
5 (most deprived)	1774 (20.0)	1686 (19.9)	88 (21.2)
Unknown	2 (0.02)	1 (0.1)	1 (0.2)
**Clinical features**			
IHD, stroke, or TIA	3177 (35.8)	2837 (33.5)	340 (81.7)
Diabetes or CKD	4389 (49.4)	4118 (48.6)	271 (65.1)
Upper GI bleeding	111 (1.2)	105 (1.2)	6 (1.4)
PPI/H2RA	—	—	351 (84.4)
DAT prescribed >12 months	—	—	274 (65.9)
**Systolic blood pressure, mmHg**			
<140	6682 (75.2)	6356 (75.1)	326 (78.4)
≥140	1262 (14.2)	1175 (13.9)	87 (20.9)
Missing	337 (3.8)	334 (3.9)	3 (0.7)
**Smoking status**			
Never smoked	3944 (44.4)	3765 (44.5)	179 (43.0)
Ex-smoker	2742 (30.9)	2602 (30.7)	140 (33.7)
Current smoker	792 (8.9)	737 (8.7)	55 (13.2)
Unknown	1403 (15.8)	1361 (16.1)	42 (10.1)
**BMI, kg/m^2^**			
<20	725 (8.2)	693 (8.2)	32 (7.7)
20–29	3733 (42.0)	3559 (42.0)	174 (41.8)
≥30	3002 (33.8)	2832 (33.5)	170 (40.9)
Unknown	1421 (16.0)	1381 (16.3)	40 (9.6)
Median (IQR)	28.4 (24.6–32.9)	28.3 (24.6–32.9)	29.3 (25.5–33.4)

^a^Ethnicity not stated or missing. AF = atrial fibrillation. BMI = body mass index. CKD = chronic kidney disease. DAT = dual antithrombotic therapy. GI = gastrointestinal. H2RA = H2-receptor antagonist. IHD = ischaemic heart disease. IMD = Index of Multiple Deprivation. IQR = interquartile range. PPI = proton pump inhibitor. TIA = transient ischaemic attack.

A total of 416 (4.7%) patients were on DAT. The majority had documented CVD (*n* = 340/416, 81.7%). Patients on DAT had substantially poorer cardiometabolic profile and more demographic and other vascular risk factors than the rest of the cohort. Among those on DAT, 65.9% (*n* = 274/416) were issued prescriptions for >12 months. Among those on DAT, 84.4% (*n* = 351/416) were on gastroprotection therapies; the proportion of these patients on DAT with a history of UGIB did not appear markedly different to the rest of the AF cohort not on DAT (1.4% [*n* = 6/416] versus 1.2% [*n* = 105/8465]) (Table 1).

### DAT

In fully adjusted multivariable logistic regression models (Table 2), male sex, younger age, South Asian ethnic group, CVD, diabetes or CKD, current smoking, and obesity (BMI ≥30 kg/m^2^) were all independently associated with greater likelihood of DAT. Individuals with CVD were over 8-fold (OR 8.01; 95% CI = 6.17 to 10.39; *P*<0.001) more likely to receive DAT compared with those without CVD. Independent of all other factors, females with AF were significantly less likely to receive DAT than males (OR 0.61; 95% CI = 0.49 to 0.77; *P*<0.001), as were older individuals (OR 0.79; 95% CI = 0.63 to 0.98; *P* = 0.032) compared with younger patients.

**Table 2.** Logistic regression models estimating the association of patient-related factors with DAT prescription among individuals with AF

	Univariable		Multivariable	
**Characteristic**	**OR (95% CI)**	***P*** **value**	**OR (95% CI)**	***P*** **value**
**Sex**				
Male	Reference	Reference	Reference	Reference
Female	0.58 (0.47 to 0.71)	**<0.001**	0.61 (0.49 to 0.77)	**<0.001**
**Age group, years**				
18–74	Reference	Reference	Reference	Reference
≥75	0.98 (0.80 to 1.19)	0.808	0.79 (0.63 to 0.98)	**0.032**
**Ethnicity**				
White	Reference	Reference	Reference	Reference
South Asian	2.32 (1.80 to 2.98)	**<0.001**	1.80 (1.37 to 2.37)	**<0.001**
Black	1.05 (0.77 to 1.44)	0.765	1.04 (0.75 to 1.45)	0.814
Unknown^a^	1.34 (0.98 to 1.82)	0.065	1.43 (1.03 to 1.99)	0.032
**IMD quintile^b^**				
1 (least deprived)	Reference	Reference	Reference	Reference
2	1.20 (0.86 to 1.68)	0.274	0.97 (0.69 to 1.37)	0.866
3	1.40 (1.01 to 1.93)	0.044	1.14 (0.81 to 1.59)	0.460
4	1.42 (1.03 to 1.96)	0.033	1.20 (0.85 to 1.67)	0.298
5 (most deprived)	1.36 (0.98 to 1.88)	0.067	1.10 (0.78 to 1.55)	0.579
**Comorbidities**				
IHD, stroke, or TIA	8.87 (6.89 to 11.42)	**<0.001**	8.01 (6.17 to 10.39)	**<0.001**
Diabetes or CKD	1.97 (1.61 to 2.42)	**<0.001**	1.42 (1.14 to 1.78)	**0.002**
Upper GI bleeding	1.17 (0.51 to 2.67)	0.718	0.86 (0.37 to 2.02)	0.736
**Systolic blood pressure, mmHg**				
<140	Reference	Reference	Reference	Reference
≥140	0.96 (0.75 to 1.22)	0.713	0.98 (0.76 to 1.26)	0.882
Unknown	0.18 (0.06 to 0.55)	**0.003**	0.50 (0.15 to 1.64)	0.252
**Smoking status**				
Never smoked	Reference	Reference	Reference	Reference
Ex-smoker	1.13 (0.90 to 1.42)	0.285	0.98 (0.76 to 1.25)	0.870
Current smoker	1.60 (1.15 to 2.15)	**0.005**	1.45 (1.03 to 2.04)	**0.032**
Unknown	0.65 (0.46 to 0.91)	0.013	1.06 (0.72 to 1.56)	0.760
**BMI, kg/m^2^**				
<20	0.94 (0.64 to 1.39)	0.772	1.03 (0.69 to 1.53)	0.894
20–29	Reference	Reference	Reference	Reference
≥30	1.23 (1.00 to 1.53)	0.064	1.38 (1.09 to 1.74)	**0.007**
Unknown	0.59 (0.42 to 0.84)	0.003	0.99 (0.67 to 1.47)	0.971

^a^Ethnicity not stated or missing. ^b^Patients with missing IMD data were excluded from the univariate logistic regression model. Bold indicates a statistically significant value. AF = atrial fibrillation. BMI = body mass index. CKD = chronic kidney disease. DAT = dual antithrombotic therapy. GI = gastrointestinal. IHD = ischaemic heart disease. IMD = Index of Multiple Deprivation. OR = odds ratio. TIA = transient ischaemic attack.

### Prolonged DAT

Most patients with AF on DAT had a record of dual prescriptions for >12 months (*n* = 274/416, 65.9%). Patient characteristics stratified by DAT duration are displayed in Supplementary Table S2. Among those on prolonged DAT, there was a disproportionate number of males (73.0% [*n* = 200/274] versus 60.6% [*n* = 86/142]), younger individuals (53.6% [*n* = 147/274] versus 49.3% [*n* = 70/142]), and individuals from South Asian ethnic backgrounds (24.5%% [*n* = 67/274] versus 18.3% [*n* = 26/142]). Those with CVD were more likely to be on prolonged DAT (85.0% [*n* = 233/274] versus 75.4% [*n* = 107/142]). The rates of UGIB did not differ among those on prolonged DAT compared with those on shorter DAT durations (1.5% [*n* = 4/274] versus 1.4% [*n* = 2/142]). In multivariable linear regression models (Table 3), prescription of gastroprotection therapies (PPI or H2RAs) was significantly associated with DAT duration of >12 months (OR 1.80; 95% CI = 1.01 to 3.22; *P* = 0.048). Apart from this, statistically significant associations were not observed between any of the other exposures and DAT duration in univariable or multivariable models.

**Table 3.** Logistic regression models estimating the association of patient-related factors associated with DAT duration >12 months (versus <6 months) in individuals with AF

	Univariable		Multivariable	
**Characteristic**	**OR (95% CI)**	***P*** **value**	**OR (95% CI)**	***P*** **value**
**Sex**				
Male	Reference	Reference	Reference	Reference
Female	0.57 (0.37 to 0.87)	**0.010**	0.63 (0.39 to 1.02)	0.060
**Age group, years**				
18–74	Reference	Reference	Reference	Reference
≥75	0.84 (0.56 to 1.26)	0.399	0.97 (0.61 to 1.55)	0.907
**Ethnicity**				
White	Reference	Reference	Reference	Reference
South Asian	1.29 (0.76 to 2.19)	0.350	1.39 (0.76 to 2.55)	0.285
Black	0.67 (0.35 to 1.25)	0.208	0.88 (0.44 to 1.75)	0.706
Unknown^a^	0.74 (0.40 to 1.37)	0.337	0.81 (0.41 to 1.62)	0.557
**IMD quintile^b^**				
1 (least deprived)	Reference	Reference	Reference	Reference
2	0.99 (0.52 to 1.89)	0.985	1.15 (0.58 to 2.29)	0.689
3	0.68 (0.35 to 1.30)	0.239	0.77 (0.38 to 1.53)	0.452
4	0.90 (0.47 to 1.72)	0.740	1.06 (0.53 to 2.13)	0.860
5 (most deprived)	0.90 (0.47 to 1.72)	0.740	0.95 (0.48 to 1.88)	0.874
**Comorbidities**				
IHD, stroke, or TIA	1.86 (1.12 to 3.08)	**0.016**	1.57 (0.91 to 2.71)	0.103
Diabetes or CKD	0.77 (0.50 to 1.18)	0.234	0.70 (0.43 to 1.15)	0.163
Upper GI bleeding	**—**	—	**—**	—
PPI/H2RA	1.83 (1.07 to 3.13)	**0.027**	1.80 (1.01 to 3.22)	**0.048**
**Systolic blood pressure, mmHg**				
<140	Reference	Reference	Reference	Reference
≥140	0.97 (0.59 to 1.59)	0.897	0.98 (0.58 to 1.67)	0.947
Unknown^c^	0.25 (0.02 to 2.84)	0.266	0.45 (0.03 to 6.47)	0.554
**Smoking status**				
Never smoked	Reference	Reference	Reference	Reference
Ex-smoker	1.39 (0.86 to 2.24)	0.175	1.21 (0.70 to 2.07)	0.497
Current smoker	1.05 (0.56 to 1.99)	0.870	1.91 (0.45 to 1.82)	0.786
Unknown	0.67 (0.34 to 1.33)	0.255	0.74 (0.32 to 1.66)	0.461
**BMI, kg/m^2^**				
<20	1.56 (0.64 to 3.84)	0.329	1.65 (0.64 to 4.24)	0.301
20–29	Reference	Reference	Reference	Reference
≥30	0.74 (0.48 to 1.16)	0.196	0.83 (0.50 to 1.37)	0.462
Unknown	0.48 (0.24 to 0.97)	0.042	0.66 (0.29 to 1.52)	0.327

^a^Ethnicity not stated or missing. ^b^Patients with missing IMD data were excluded from the univariate logistic regression model. ^c^Patients with missing systolic blood pressure data were excluded from the univariate logistic regression model. Bold indicates a statistically significant value. AF = atrial fibrillation. BMI = body mass index. CKD = chronic kidney disease. DAT = dual antithrombotic therapy. GI = gastrointestinal. H2RA = H2-receptor antagonist. IHD = ischaemic heart disease. IMD = Index of Multiple Deprivation. OR = odds ratio. PPI = proton pump inhibitor. TIA = transient ischaemic attack.

### Gastric protection

Among the 416 patients with AF on DAT, 84.4% (*n* = 351) were prescribed gastroprotection (PPIs or H2RAs); of these 346 (98.6%) were prescribed PPI and five (1.4%) patients were prescribed H2RA alone. The characteristics of this subset is presented in Supplementary Table S3. Those receiving gastroprotection were more likely to be female (31.9% [*n* = 112/351] versus 27.7% [*n* = 18/65]), from ethnic minority backgrounds (48.7% [*n* = 171/351] versus 35.4% [*n* = 23/65]), and to have comorbidities, including CVD, diabetes, CKD, or previous UGIB (see Supplementary Table S3).

In fully adjusted models (Table 4), those with CVD were over 3-fold more likely to receive gastroprotection (OR 3.33; 95% CI = 1.71 to 6.47; *P*<0.001), compared with those without CVD. Similarly, independent of all other exposures, individuals from a South Asian ethnic group were significantly more likely to receive PPI or H2RA prescriptions (OR 2.70; 95% CI = 1.15 to 6.32; *P* = 0.023) compared with those from White ethnic backgrounds.

**Table 4.** Logistic regression models estimating the association of patient-related factors associated with PPI/H2RA prescription in individuals with AF on DAT

	Univariable		Multivariable	
**Characteristic**	**OR (95% CI)**	***P*** **value**	**OR (95% CI)**	***P*** **value**
**Sex**				
Male	Reference	Reference	Reference	Reference
Female	1.22 (0.68 to 2.20)	0.501	1.33 (0.68 to 2.56)	0.403
**Age group, years**				
18–74	Reference	Reference	Reference	Reference
≥75	1.01 (0.59 to 1.71)	0.980	0.84 (0.45 to 1.58)	0.588
**Ethnicity**				
White	Reference	Reference	Reference	Reference
South Asian	2.18 (1.01 to 4.68)	0.046	2.70 (1.15 to 6.32)	**0.023**
Black	1.20 (0.52 to 2.74)	0.672	1.46 (0.59 to 3.65)	0.416
Unknown^a^	1.79 (0.72 to 4.46)	0.213	3.30 (1.13 to 9.66)	0.029
**IMD quintile^b^**				
1 (least deprived)	Reference	Reference	Reference	Reference
2	0.65 (0.28 to 1.48)	0.301	0.54 (0.22 to 1.30)	0.170
3	1.15 (0.45 to 2.96)	0.765	1.42 (0.52 to 3.89)	0.489
4	0.82 (0.35 to 1.96)	0.659	0.69 (0.27 to 1.75)	0.434
5 (most deprived)	0.75 (0.32 to 1.77)	0.516	0.65 (0.26 to 1.63)	0.362
**Comorbidities**				
IHD, stroke, or TIA	2.34 (1.29 to 4.26)	**0.005**	3.33 (1.71 to 6.47)	**<0.001**
Diabetes or CKD	1.40 (0.82 to 2.41)	0.220	1.33 (0.71 to 2.47)	0.370
Upper GI bleeding	**—**	—	**—**	—
**Systolic blood pressure, mmHg**				
<140	Reference	Reference	Reference	Reference
≥140	2.04 (0.94 to 4.48)	0.072	2.07 (0.90 to 4.74)	0.087
Unknown^c^	**—**	—	**—**	—
**Smoking status**				
Never smoked	Reference	Reference	Reference	Reference
Ex-smoker	1.23 (0.68 to 2.25)	0.494	1.69 (0.84 to 3.31)	0.144
Current smoker	1.78 (0.70 to 4.50)	0.225	1.88 (0.69 to 5.14)	0.217
Unknown	1.31 (0.51 to 3.36)	0.580	2.32 (0.72 to 7.46)	0.157
**BMI, kg/m^2^**				
<20	1.00 (0.35 to 2.80)	0.988	1.13 (0.36–3.52)	0.833
20–29	Reference	Reference	Reference	Reference
≥30	1.02 (0.57 to 1.83)	0.954	1.14 (0.58 to 2.26)	0.702
Unknown	0.87 (0.35 to 2.16)	0.757	0.96 (0.32 to 2.91)	0.946

^a^Ethnicity not stated or missing. ^b^Patients with missing IMD data were excluded from the univariate logistic regression model. ^c^Patients with missing systolic blood pressure data were excluded from the univariate logistic regression model. Bold indicates a statistically significant value. AF = atrial fibrillation. BMI = body mass index. CKD = chronic kidney disease. DAT = dual antithrombotic therapy. GI = gastrointestinal. H2RA = H2-receptor antagonist. IHD = ischaemic heart disease. IMD = Index of Multiple Deprivation. OR = odds ratio. PPI = proton pump inhibitor. TIA = transient ischaemic attack.

## Discussion

### Summary

In this large population-based cohort of 8881 patients with AF, 4.7% (*n* = 416) were on DAT. Male sex, younger age, South Asian ethnic group, CVD, diabetes or CKD, current smoking, and obesity were all independently associated with greater likelihood of DAT. Independent of all other factors, female and older patients with AF were significantly less likely to receive DAT than male and younger patients. Among patients with AF on DAT, almost two-thirds (65.9%, *n* = 274/416) received prescriptions for >12 months, which indicates an inappropriately prolonged duration. Greater use of PPI or H2RA was the only factor significantly linked to prolonged DAT prescription. Among patients with AF on DAT, 84.4% (*n* = 351/416) were on gastroprotective therapies (PPI or H2RAs). The presence of CVD (IHD, stroke, or TIA) was associated with greater likelihood of PPI or H2RA.

### Strengths and limitations

This large-scale epidemiologic study of routine primary care health data allowed examination of treatment practices in an ethnically diverse population cohort. The use of standardised disease codes and complete prescription records supported reliable ascertainment of case and treatment status across a large, unselected sample. Use of routine data may have some limitations. Ascertainment of AF cases may be limited by misdiagnoses or coding errors. Furthermore, the study defined DAT and PPI or H2RA use based on primary care prescription records, without assessment of medication adherence. It is also possible that a small minority of patients were using medications obtained from sources other than primary care or were using medications issued outside of the analysis window. Such individuals would not be captured in the study, but are unlikely to be in numbers to appreciably alter the results. It was assumed that concurrent prescription of medications indicates concurrent usage. While for most people this would be correct, it is possible that some people were categorised as DAT users but had in fact discontinued their antiplatelet medicine. The definition of prolonged DAT (>12 months) does not account for individuals who may have stopped and restarted DAT for an appropriate indication. Finally, very uncommonly, extended DAT may be appropriate. Patient records were unable to be viewed individually and patients on appropriate prolonged DAT or who received DAT prescriptions but did not actually take the medication could not be distinguished. However, the number of such individuals is likely to be small and would not explain the large proportion of patients on prolonged DAT in the cohort (*n* = 274, 65.9% of those on DAT). The study did not consider the further duration of therapy beyond 12 months as for the vast majority this is clearly an excessive duration. Future work considering more granular risk would be informative, but information electronically coded in UK GP records is limited to the issue of a prescription and there is no adequate record of patients not completing prescriptions.

There are many factors that may influence bleeding risk. This article aimed to provide a broad characterisation of individuals who were prescribed DAT; the clinical factors considered in this study are not exhaustive. A key outcome in evaluating DAT is the frequency of UGIB. The few UGIB events in the sample precluded comparisons with this outcome. Finally, the sample from east London has greater ethnic diversity, deprivation, and comorbidities than the national UK population. Similar evaluations at a national level would provide further insight on the topic.

### Comparison with existing literature

The risk factors associated with DAT in the study are consistent with previous reports.^19–22^ Among patients with AF on DAT, 81.7% (*n* = 340/416) had co-existent CVD (IHD, stroke, or TIA). There were 76 patients (18.3%) who were on DAT, but without documented CVD; it was noted that these individuals were significantly less likely to receive PPI or H2RA than those with record of CVD. It is possible that CVDs were present but are not correctly coded for these patients or that they have alternative, less common indications for antiplatelet therapy such as peripheral arterial disease or haematological diseases. Either way, the findings suggest that for these patients the indication for DAT was unclear and associated with lower rates of gastroprotection prescription.

A record of DAT was found in 4.7% (*n* = 416) of the study sample. This is lower than in previous studies and indicates a decreasing contemporary trend when compared with other nationwide studies dating from >10 years earlier. Previous reports from UK data between 1998 and 2010 indicate that 11% of patients with AF and IHD were on DAT.^23^ In a similar population of patients with AF and IHD between 2010 and 2011, rates of DAT were reported at 27%.^24^ Corresponding figures from Japan^25^ (2016–2018) and Italy^26^ (2018–2019) are 15.1% and 8.8%, respectively. A key difference between the present studies and these reports is that the latter are all limited to individuals with AF and IHD, in whom guideline recommendations for DAT are generally well-defined. The current study included all patients with those with AF and as such a lower proportion on DAT would be expected. Other potential explanations include differences in study populations, more contemporary results, and differences in national practices.

Among patients with AF on DAT, 84.4% (*n* = 351) were prescribed a PPI or H2RA, which is higher than reported by other cohort studies (11%–63%).^25,27,28^ A relatively high figure was achieved possibly by virtue of a local incentive programme promoting the use of PPI optimisation, or simply because contemporary guidance and research is more likely to endorse the use of PPI co-therapy with antithrombic medicines. Indeed, a recent European Society of Cardiology guidance update highlighted bleeding risk reduction as a key priority area, including recommendation for routine use of PPI in combined uses of antiplatelet agents with either another antiplatelet drug or an anticoagulant.^6^ As indicated in the above discussion, prescription of DAT was significantly higher in patients with documented CVD, indicating that perhaps these patients had clearer healthcare plans.

Independent of all other demographic and clinical factors, women with AF had significantly lower rates of DAT prescription than males (5.7% versus 3.4%), which may suggest undertreatment of women. Previous work has demonstrated underdiagnosis,^29^ undertreatment, and delayed treatment of women with CVD compared with men.^30,31^ Poorer risk factor profile and IHD patterns associated with higher thrombotic risk^32–36^ were not evaluated in the current study, and may be driving greater use of DAT in males. Additionally, greater perception of bleeding risk in women may be driving a reluctance for DAT in this cohort. Indeed, among women prescribed DAT, greater use of gastric protection therapies were observed than in men (31.9% versus 27.3%), although this difference was not statistically significant. There are likely to be other factors influencing sex-differential practices, which would be a subject for further research.

### Implications for research and practice

The findings underline several key management considerations. Among patients with AF, 4.7% were receiving DAT, of these, over 65% were prescribed DAT for inappropriately prolonged durations. This highlights the importance of clear documentation of indications for DAT and its duration, with a requirement for continuing regular review of any DAT use beyond 6 months. Although higher rates of PPI or H2RA prescription in those on prolonged DAT were observed, further research is indicated to investigate the efficacy of PPI/H2RA in prolonged durations of DAT. Second, 16% of patients on DAT were not prescribed PPI or H2RA. Gastroprotection therapy should be routinely recommended for older patients receiving multiple antithrombotic agents or those with other UGIB risk factors.^37,38^ Finally, it was observed that woemn were significantly less likely to receive DAT, even when all other patient-related factors were accounted for; possible undertreatment of females warrants further study.

## Data

The dataset relied on in this article is available from the corresponding author on reasonable request.
